# Comparison of different treatment strategies for T3N1-3 stage gastric cancer based on the SEER database

**DOI:** 10.1038/s41598-024-61904-8

**Published:** 2024-05-16

**Authors:** Yimei Tan, Shuanghua Liu, Shaohong Tao, Hui Cheng, Menghe Huang, Qizhi Tang

**Affiliations:** 1 Affiliated Guangdong Hospital of Integrated Traditional Chinese and Western Medicine of Guangzhou University of Chinese Medicine, No.16, Guicheng South Fifth Road, Foshan, 528200 Guangdong China; 2 Guangdong Provincial Hospital of Integrated Traditional Chinese and Western Medicine, No.16, Guicheng South Fifth Road, Foshan, 528200 Guangdong China; 3 Jinan University, No.601, Huangpu Avenue West, Guangzhou, 510632 Guangdong China

**Keywords:** Gastric cancer, Clinical stage T3N1-3, Treatment options, Seer database, Cancer, Medical research, Oncology

## Abstract

Treatment options for T3N1 stage gastric cancer exhibit regional variation, with optimal approach remaining unclear. We derived our data from the SEER database, using Cox proportional risk regression models for univariate and multivariate analyses of 5-years overall survival (5yOS) and 5-years cancer-specific survival (5yCSS). The results showed that younger age, female, non-white race, highly differentiated histologic grade, non-Signet ring cell adenocarcinoma, low N stage, lesser curvature of the stomach, OP followed by adjuvant C/T with or without RT, partial gastrectomy, C/T and others, Radiation therapy, and Chemotherapy were significantly associated with better 5yOS and 5yCSS. For patients with stage T3N1-3 gastric cancer, multimodal treatment regimens demonstrate superior survival outcomes compared to surgery or radiotherapy alone. Among them, OP followed by adjuvant C/T with or without RT emerges as particularly efficacious, potentially offering enhanced benefits for non-Asian populations.

## Introduction

Gastric cancer (GC) is not only the fifth most prevalent cancer in the world, with more than 1 million new cases each year, but also the fourth leading cause of cancer death, according to the latest global cancer burden data for 2020 released by the International Agency for Research on Cancer (IARC) of the World Health Organization^1,2^. With the advancement of diagnosis and treatment technology, the 5-years survival rate of early gastric cancer patients has exceeded 90%^3^. Nonetheless, survival outcomes hinge significantly on tumor staging, with locally advanced gastric cancer prognosis remaining grim due to its aggressive nature and swift progression^4^. Moreover, variations in tumor biological characteristics, patient demographics, and treatment strategies contribute to significant disparities in the 5-years survival rates of gastric cancer patients across different regions^5–7^.

Strategies for the treatment of locally advanced gastric cancer aimed at improving patient survival and requiring expertise from multiple disciplines, and multidisciplinary treatment decisions are made after a comprehensive evaluation by specialists in oncology, surgery, radiotherapy, imaging, and pathology^4,7^. Several regional treatment guidelines recommend radical gastrectomy combined with neoadjuvant chemotherapy, perioperative chemotherapy, and postoperative adjuvant chemotherapy (with or without radiotherapy) for patients with locally advanced gastric cancer^8–10^. Recent studies have revealed the possibility of modulating cellular composition and function within the tumor microenvironment to influence gastric cancer growth, metastasis, and survival outcomes. Nonetheless, the limited overall therapeutic efficacy, significant inter-individual variability in response, and safety concerns associated with immunotherapy have impeded its widespread adoption and utilization^11–13^. Currently, there are numerous treatment options for stage T3N1-3 gastric cancer, however, decisions on treatment regimens vary somewhat by region, and the optimal treatment remains unclear^14–16^. Through a retrospective analysis of the SEER database 17 to compare the overall and specific survival rates of different treatment modalities for T3N1-3 gastric cancer, thereby discerning the optimal therapeutic approach for T3N1-3 gastric cancer.

## Methods

Our research materials were derived from demographics, tumor characteristics, treatment regimens, and outcome data for patients with gastric cancer diagnosed between 2010 and 2015 from the National Cancer Institute's Surveillance, Epidemiology, and End Results (SEER) database. Study data were extracted from the SEER 17 registry database using SEER*Stat software (version 8.4.2). The study was conducted using de-identified medical information data from the SEER database and therefore no ethics committee approval and informed consent from patients was required.

### Study population

Participants meeting the following criteria were included in this study: (1) All patients were diagnosed with gastric cancer in 2010–2015. (2) Age at diagnosis > 18 years. (3) Compliance with AJCC Cancer Staging Manual 7th edition TNM staging of T3, N1-3, M0. (4) No other type of cancer diagnosed in a lifetime. Participants were excluded if (1) Survival months were 0 or unknown. (2) Neither surgical treatment nor radiotherapy was received. (3) Not histologically confirmed. (4) Gastric cancer is not the first malignant tumor.

### Data extraction

Extracted data included sex, age at diagnosis, race record, marital status at diagnosis, primary site, ICD-O-3 histology code and behavior, CS tumor size, CS lymph nodes, Regional nodes positive, Regional nodes examined, pathologic grade, derived AJCC T and N stages, radiation sequence with surgery, systemic therapy sequence with surgery, RX Summary-Surgery Primary Site, radiation recode, chemotherapy recode, survival months, vital status recode, COD to site recode, SEER cause-specific death classification.

### Statistical analysis

Statistical analysis and plotting were performed using R language (version 4.3.1). Continuous variables were converted to categorical variables, and the chi-square test was used to compare the clinicopathologic characteristics of gastric cancer patients with different N-stages. Overall survival (OS) was defined as the time from the month of diagnosis to death from any cause or the last follow-up. Cancer-specific survival (CSS) was defined as cancer death, that is, the time from the month of diagnosis to death from gastric cancer or the last follow-up visit. Survival analysis was performed by plotting survival curves using the Kaplan–Meier method, and differences in CSS and OS between variables were compared by the log-rank test, and *P* values were determined. Univariate and multivariate analyses of CSS and OS were conducted using the Cox proportional risk regression model. *P* < 0.05 indicated that the difference was statistically significant.

## Results

**Figure 1:**
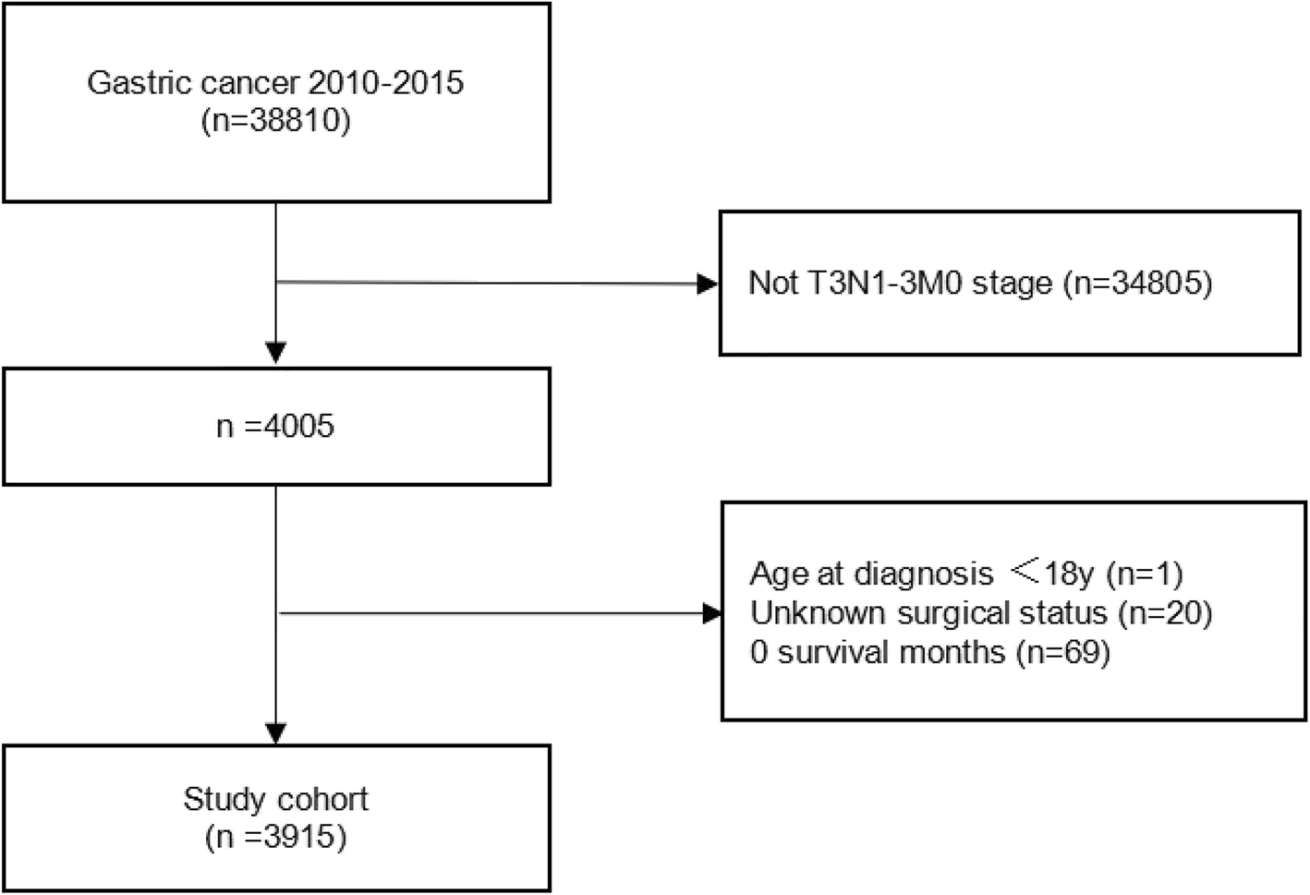
Patient selection.

Ultimately, 3915 patients with T3N1-3M0 gastric cancer were enrolled in this study, with the study population screening process depicted in Fig. 1. Among them, 2013 patients were in the T3N1M0 stage, 1103 with T3N2M0 stage, and 799 with T3N3M0 stage (refer to Table 1 for details). The general characteristics of the population of 3915 gastric cancer patients included in this study were skewed toward ≥ 60 years of age (N1: 1452 [72.13%]; N2:755 [68.45%]; N3: 525 [65.71%]), male (N1: 1479 [73.47%]; N2: 810 [73.44%]; N3: 554 [69.34%]), white (N1: 1576 [78.29%]; N2: 804 [72.89%]; N3: 530 [66.33%]), and married (N1: 1247 [61.95%]; N2: 708 [64.19%]; N3: 513 [64.21%]).

**Table 1:** Baseline characteristics.

Parameters	T3N1 (n = 2013)	T3N2 (n = 1103)	T3N3 (n = 799)	*P* Value
Age at diagnosis, n (%)				0.003
< 40	48 (2.38)	30 (2.72)	33 (4.13)	
40–60	513 (25.48)	318 (28.83)	241 (30.16)	
≥ 60	1452 (72.13)	755 (68.45)	525 (65.71)	
Sex, n (%)				0.066
Female	534 (26.53)	293 (26.56)	245 (30.66)	
Male	1479 (73.47)	810 (73.44)	554 (69.34)	
Race, n (%)				< 0.001
White	1576 (78.29)	804 (72.89)	530 (66.33)	
Black	193 (9.59)	136 (12.33)	98 (12.27)	
Others	244 (12.12)	163 (14.78)	171 (21.40)	
Marital Status, n (%)				0.068
Unmarried, single	292 (14.51)	168 (15.23)	109 (13.64)	
Married	1247 (61.95)	708 (64.19)	513 (64.21)	
Previously married	404 (20.07)	176 (15.96)	140 (17.52)	
Unknown	70 (3.48)	51 (4.62)	37 (4.63)	
Tumor location, n (%)				< 0.001
Body	98 (4.87)	84 (7.62)	71 (8.89)	
Cardia	1199 (59.56)	495 (44.88)	242 (30.29)	
Fundus	54 (2.68)	37 (3.35)	26 (3.25)	
Gastric antrum	266 (13.21)	189 (17.14)	175 (21.90)	
Greater	50 (2.48)	32 (2.90)	31 (3.88)	
Lesser	134 (6.66)	112 (10.15)	91 (11.39)	
Pylorus	39 (1.94)	32 (2.90)	31 (3.88)	
Others	173 (8.59)	122 (11.06)	132 (16.52)	
Histology, n (%)				< 0.001
Signet ring cell adenocarcinoma	267 (13.26)	196 (17.77)	172 (21.53)	
Adenocarcinoma,intestinal type	203 (10.08)	142 (12.87)	111 (13.89)	
Adenocarcinoma, diffuse type	76 (3.78)	55 (4.99)	75 (9.39)	
Mucinous adenocarcinoma	36 (1.79)	24 (2.18)	24 (3.00)	
Adenocarcinoma, NOS	1209 (60.06)	592 (53.67)	354 (44.31)	
Others	222 (11.03)	94 (8.52)	63 (7.88)	
Grade, n (%)				< 0.001
I	75 (3.73)	18 (1.63)	15 (1.88)	
II	574 (28.51)	266 (24.12)	128 (16.02)	
III	1132 (56.23)	714 (64.73)	603 (75.47)	
IV	35 (1.74)	27 (2.45)	25 (3.13)	
Unknown	197 (9.79)	78 (7.07)	28 (3.50)	
Tumor size, n (%)				< 0.001
< 5 cm	877 (43.57)	470 (42.61)	306 (38.30)	
≥ 5 cm	678 (33.68)	459 (41.61)	433 (54.19)	
Unknown	458 (22.75)	174 (15.78)	60 (7.51)	
Therapy group, n (%)				< 0.001
OP only	219 (10.88)	185 (16.77)	194 (24.28)	
OP + adjuvant C/T ± RT	286 (14.21)	280 (25.39)	352 (44.06)	
CRRT + OP	425 (21.11)	176 (15.96)	43 (5.38)	
Neoadjuvant C/T + OP + adjuvant C/T ± RT	171 (8.49)	116 (10.52)	73 (9.14)	
C/T and others	912 (45.31)	346 (31.37)	137 (17.15)	
Surgical therapy, n (%)				< 0.001
No surgery	696 (34.58)	209 (18.95)	47 (5.88)	
Partial gastrectomy	858 (42.62)	574 (52.04)	454 (56.82)	
Total (or near-total) gastrectomy	459 (22.80)	320 (29.01)	298 (37.30)	
Radiation, n (%)				< 0.001
Yes	1197 (59.46)	579 (52.49)	381 (47.68)	
No/Unknown	816 (40.54)	524 (47.51)	418 (52.32)	
Chemotherapy, n (%)				< 0.001
Yes	1642 (81.57)	875 (79.33)	580 (72.59)	
No/Unknown	371 (18.43)	228 (20.67)	219 (27.41)	
Overall survival rate, %				< 0.001
1y	73.1	73.3	65.8	
3y	40.9	37.4	26.7	
5y	31.2	25.9	17.6	

The oncological characteristics of the gastric cancer patients in this study were as follows. In terms of tumor location, Cardia (N1: 59.56%; N2: 44.88%; N3: 30.29%) and Gastric antrum (N1: 13.21%; N2: 17.14%; N3: 21.90%) were predominant. Tumor histology was dominated by Adenocarcinoma, NOS (N1: 60.06%; N2: 53.67%; N3: 44.31%) and Signet ring cell adenocarcinoma (N1: 13.26%; N2: 17.77%; N3: 21.53%). Histologic grading mainly consisted of poorly differentiated (N1: 56.23%; N2: 64.73%; N3: 75.47%) and moderately differentiated (N1: 28.51%; N2: 24.12%; N3: 16.02%). Notably, the proportion of tumors with a size ≥ 5cm increased progressively with advancing N stage, correlating with a heightened risk of lymph node metastasis.

Variations in treatment selections among gastric cancer patients across different N stages were observed in this study. Predominantly, patients in N1 stage favored adjuvant chemotherapy (C/T) and others (45.31%), while a notable proportion opted for concurrent chemoradiotherapy (CCRT) combined with operation (OP) (21.11%). A total of 31.37% of patients with stage N2 gastric cancer opted to receive C/T and others, whereas 25.39% received OP followed by adjuvant C/T with or without radiotherapy (RT). The majority of N3-stage patients received OP followed by adjuvant C/T with or without RT (44.06%) or OP alone (24.28%). The likelihood of surgical intervention increased with escalating N stage, with partial gastrectomy emerging as the most frequently chosen surgical modality (N1: 42.62%; N2: 52.04%; N3: 56.82%). Chemotherapy was administered to the majority of patients (N1: 81.57%; N2: 79.33%; N3: 72.59%), with minimal disparity observed in the proportion of patients with T3N1-3M0 gastric cancer receiving radiotherapy compared to those who did not.

There were significant differences in the 1-, 3-, and 5-years overall survival rates between N1-N3 stages. The 1yOS was above 60% in all N1-N3 patients, but the 5yOS was lower and exhibited considerable variability (N1: 31.2%; N2: 25.9%; N3: 17.6%). The 5yOS and 5yCSS of the multidisciplinary treatment modalities were significantly better than those of OP alone and C/T and other treatment modalities.

**Figure 2:**
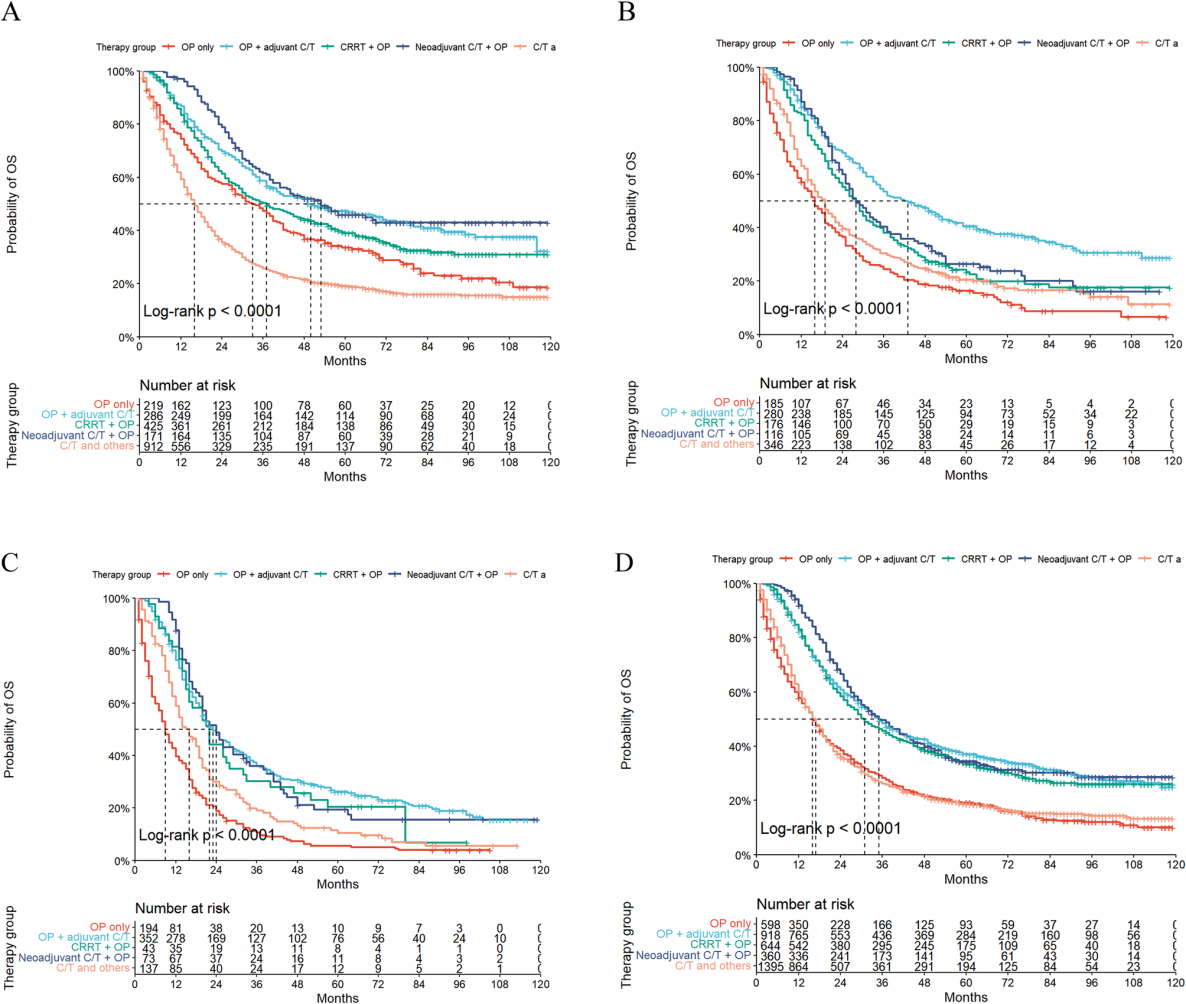
Kaplan–Meier survival curves show 5yOS rate for T3 gastric cancer patients with different N-stages based on different treatment options. (**A**)T3N1, (**B**)T3N2, (**C**)T3N3, (**D**)T3N1-3.

**Figure 3:**
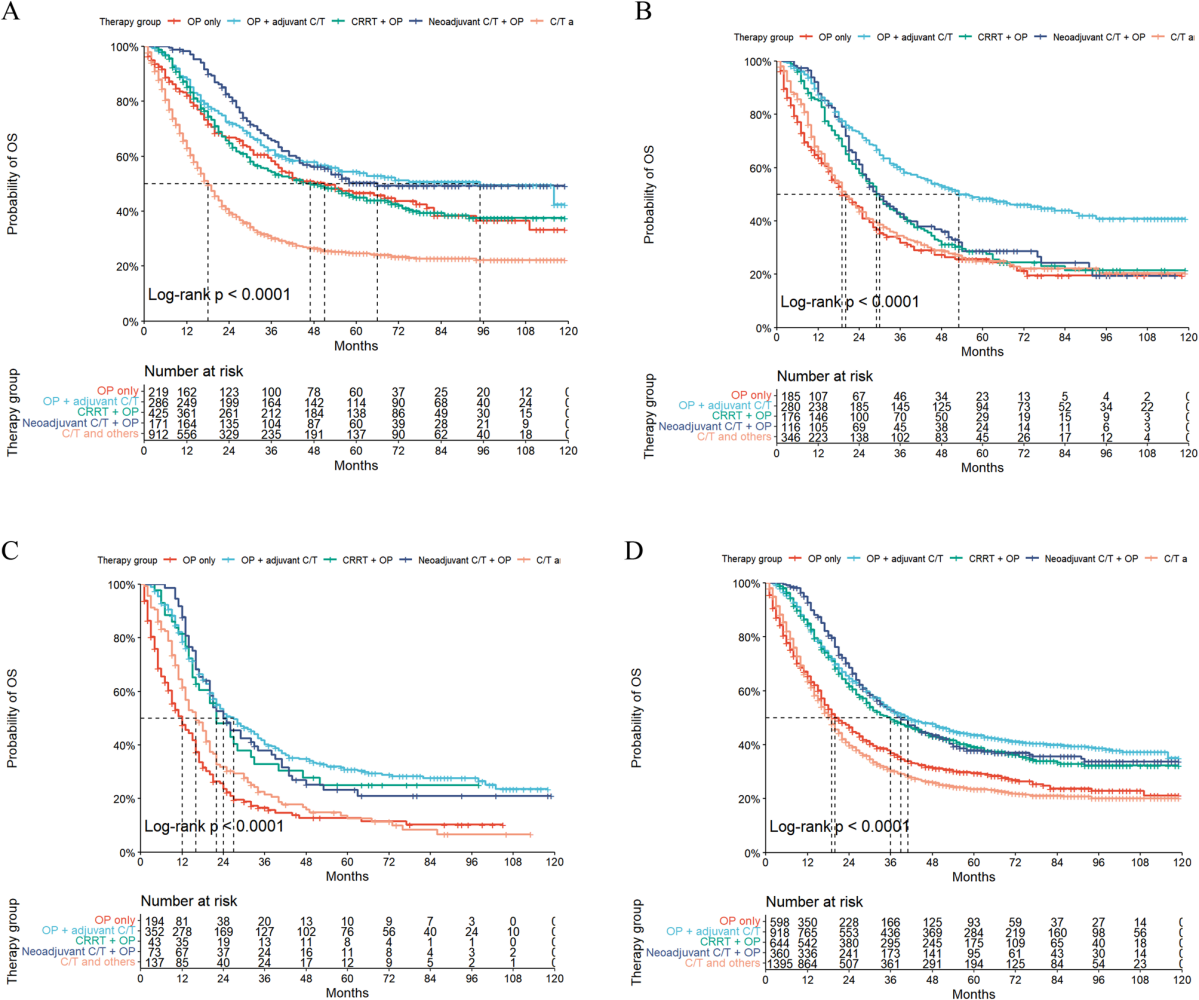
Kaplan–Meier survival curves show 5yCSS rate for T3 gastric cancer patients with different N-stages based on different treatment options. (**A**)T3N1, (**B**)T3N2, (**C**)T3N3, (**D**)T3N1-3.

A comparison of 5yOS and 5yCSS in T3N1-3 gastric cancer under different treatment regimens was performed (see Supplementary Material for details). Treatment categories included OP alone (group 1), OP followed by adjuvant C/T with or without RT (group 2), CRRT followed by OP (group 3), neoadjuvant C/T followed by OP and adjuvant C/T with or without RT (group 4), and C/T in conjunction with other modalities (group 5). There were significant differences in 5yOS and 5yCSS under different treatment regimens in patients with N1-3 stages of gastric cancer. Figures 2 and 3 displayed the 5yOS and 5yCSS survival curves based on different treatment regimens for gastric cancer patients with different N-stages. Notably, 5yOS was suboptimal, falling below 50% for N1-3 stages of gastric cancer. Overall, the 5yOS with OP only or C/T and others was lower than that of the other three treatment groups, in which the 5yOS of T3N3 gastric cancer treated with OP only was only 5%. The 5yOS was the highest for all stages of gastric cancer treated with OP combined with adjuvant C/T with or without RT, in which the 5yOS for T3N1 stage gastric cancer patients treated with neoadjuvant C/T followed by OP and adjuvant C/T with or without RT was comparable to that of OP followed by adjuvant C/T with or without RT. In contrast, the 5yCSS with OP only was comparable to multidisciplinary combination therapy in patients with T3N1 stage gastric cancer, and the difference between 5yCSS (46.6%) and 5yOS (33.6%) was greater with OP only. We speculated that it might be related to the number of regional lymph node metastases. The conclusions of 5yCSS and 5yOS for gastric cancer with different N-stages were consistent, with the rank sum test indicating significant differences between different treatments (*P* < 0.05).

A comparison of 5yOS and 5yCSS in T3N1-3 gastric cancer under different surgical therapies was performed (see Supplementary Material for details). Notably, patients who did not undergo surgery exhibited 5yCSS rates below 20%, underscoring the importance of surgical intervention. Partial gastrectomy yielded improved survival outcomes; however, notable discrepancies were observed among different N-staging groups (N1: 52.6%; N2: 37.9%; N3: 25.2%). There were significant differences between different surgical approaches (*P* < 0.05). The 5yOS and 5yCSS survival curves for different surgical approaches are shown in Supplementary Material.

Univariate and multivariate analyses using Cox proportional risk regression models indicated that younger, female, non-white race, highly differentiated histologic grade, non-Signet ring cell adenocarcinoma, low N stage, lesser curvature of the stomach, OP + adjuvant C/T ± RT, partial gastrectomy, C/T and others, Radiation therapy, and Chemotherapy were significantly associated with better 5yOS and CSS. However, previously married patients with gastric cancer have a poorer prognosis. (See Table 2 for details).

**Table 2:** Univariate and multivariate analysis of OS and CSS.

Parameters	Overall survival				Cancer-specifc survival			
	Univariate		Multivariate		Univariate		Multivariate	
	HR (95% CI)	*P* Value	HR (95% CI)	*P* Value	HR (95% CI)	*P* Value	HR (95% CI)	*P* Value
Age at diagnosis								
< 40	Ref		Ref		Ref		Ref	
40–60	1.15(0.90–1.47)	0.275	1.08(0.84–1.38)	0.534	1.13(0.88–1.47)	0.342	1.08(0.83–1.41)	0.546
≥ 60	1.54(1.21–1.95)	< 0.001	1.32(1.04–1.69)	0.024	1.39(1.08–1.78)	0.011	1.23(0.95–1.59)	0.122
Sex								
Female	Ref		Ref		Ref		Ref	
Male	1.08(0.99–1.17)	0.067	1.17(1.07–1.28)	< 0.001	1.11(1.01–1.21)	0.026	1.17(1.06–1.29)	0.001
Race								
White	Ref		Ref		Ref		Ref	
Black	0.90(0.80–1.00)	0.072	1.00(0.88–1.10)	0.985	0.86(0.75–0.98)	0.025	0.98(0.85–1.13)	0.792
Others	0.81(0.73–0.90)	< 0.001	0.89(0.79–0.99)	0.032	0.79(0.70–0.88)	< 0.001	0.88(0.78–0.99)	0.037
Marital Status								
Unmarried, single	Ref		Ref		Ref		Ref	
Married	1.02(0.91–1.13)	0.762	0.99(0.88–1.10)	0.799	1.01(0.90–1.13)	0.927	0.99(0.88–1.11)	0.833
Previously married	1.38(1.22–1.57)	< 0.001	1.25(1.10–1.43)	< 0.001	1.20(1.13–1.49)	< 0.001	1.23(1.06–1.41)	0.005
Unknown	1.13(0.92–1.38)	0.249	0.99(0.81–1.22)	0.954	1.06(0.85–1.33)	0.593	0.96(0.76–1.20)	0.710
Tumor location								
Body	Ref		Ref		Ref		Ref	
Cardia	1.14(0.98–1.33)	0.092	1.08(0.91–1.27)	0.380	1.21(1.02–1.43)	0.025	1.12(0.93–1.35)	0.221
Fundus	1.09(0.85–1.40)	0.504	0.99(0.77–1.28)	0.950	1.09(0.82–1.44)	0.543	0.99(0.75–1.32)	0.981
Gastric antrum	0.92(0.77–1.09)	0.341	0.89(0.74–1.05)	0.172	0.92(0.76–1.11)	0.376	0.90(0.74–1.09)	0.273
Greater	0.86(0.66–1.13)	0.291	0.85(0.65–1.11)	0.233	0.88(0.65–1.19)	0.402	0.87(0.65–1.18)	0.385
Lesser	0.88(0.73–1.07)	0.197	0.82(0.67–0.99)	0.039	0.85(0.68–1.05)	0.134	0.79(0.64–0.98)	0.031
Pylorus	1.18(0.91–1.53)	0.202	1.12(0.86–1.45)	0.407	1.21(0.91–1.61)	0.180	1.16(0.87–1.54)	0.310
Others	1.20(1.00–1.44)	0.045	1.03(0.86–1.23)	0.767	1.21(0.99–1.47)	0.066	1.03(0.84–1.26)	0.782
Histology								
Signet ring cell adenocarcinoma	Ref		Ref		Ref		Ref	
Adenocarcinoma,intestinal type	0.72(0.62–0.85)	< 0.001	0.76(0.66–0.89)	< 0.001	0.65(0.56–0.76)	< 0.001	0.73(0.62–0.86)	< 0.001
Adenocarcinoma, diffuse type	0.81(0.68–0.97)	0.021	0.90(0.75–1.08)	0.263	0.79(0.65–0.96)	0.016	0.88(0.73–1.08)	0.224
Mucinous adenocarcinoma	0.77(0.59–1.01)	0.061	0.89(0.68–1.17)	0.400	0.77(0.58–1.03)	0.074	0.90(0.67–1.20)	0.459
Adenocarcinoma, NOS	0.90(0.81–0.99)	0.033	0.85(0.76–0.95)	0.003	0.87(0.78–0.97)	0.011	0.82(0.73–0.92)	< 0.001
Others	0.83(0.72–0.96)	0.014	0.79(0.68–0.92)	0.002	0.76(0.65–0.90)	< 0.001	0.74(0.63–0.87)	< 0.001
Grade								
I	Ref		Ref		Ref		Ref	
II	0.92(0.74–1.16)	0.503	1.00(0.80–1.27)	0.950	1.07(0.82–1.40)	0.611	1.17(0.90–1.53)	0.240
III	1.11(0.89–1.38)	0.357	1.25(1.01–1.57)	0.049	1.29(0.99–1.67)	0.050	1.46(1.12–1.89)	0.005
IV	0.94(0.68–1.13)	0.733	1.04(0.74–1.45)	0.835	1.07(0.74–1.55)	0.727	1.18(0.81–1.72)	0.403
Unknown	1.09(0.85–1.41)	0.483	0.93(0.72–1.19)	0.551	1.30(0.98–1.74)	0.072	1.08(0.81–1.45)	0.596
Tumor size								
< 5 cm	Ref		Ref		Ref		Ref	
≥ 5 cm	1.06(0.98–1.15)	0.143	0.99(0.91–1.07)	0.779	1.08(0.99–1.18)	0.079	1.01(0.92–1.10)	0.838
Unknown	1.41(1.28–1.56)	< 0.001	1.16(1.04–1.29)	0.007	1.51(1.36–1.68)	< 0.001	1.21(1.08–1.36)	0.001
N stage								
N1	Ref		Ref		Ref		Ref	
N2	1.12(1.03–1.22)	0.011	1.30(1.20–1.42)	< 0.001	1.13(1.03–1.24)	0.010	1.34(1.22–1.47)	< 0.001
N3	1.47(1.34–1.61)	< 0.001	1.98(1.78–2.19)	< 0.001	1.51(1.36–1.66)	< 0.001	2.09(1.87–2.34)	< 0.001
Therapy group								
OP only	Ref		Ref		Ref		Ref	
OP + adjuvant C/T ± RT	0.52(0.47–0.59)	< 0.001	0.89(0.73–1.10)	0.281	0.58(0.50–0.66)	< 0.001	0.97(0.77–1.21)	0.756
CRRT + OP	0.56(0.49–0.64)	< 0.001	1.07(0.86–1.34)	0.548	0.65(0.56–0.74)	< 0.001	1.20(0.94–1.53)	0.137
Neoadjuvant C/T + OP + adjuvant C/T ± RT	0.50(0.43–0.58)	< 0.001	0.88(0.70–1.10)	0.269	0.59(0.50–0.70)	< 0.001	1.02(0.80–1.30)	0.874
C/T and others	0.96(0.87–1.07)	0.483	0.84(0.69–1.02)	0.080	1.11(0.98–1.25)	0.092	0.91(0.73–1.12)	0.368
Surgical therapy								
No surgery	Ref		Ref		Ref		Ref	
Partial gastrectomy	0.42(0.38–0.46)	< 0.001	0.32(0.28–0.37)	< 0.001	0.39(0.36–0.43)	< 0.001	0.30(0.25–0.35)	< 0.001
Total (or near-total) gastrectomy	0.49(0.44–0.54)	< 0.001	0.37(0.32–0.43)	< 0.001	0.47(0.42–0.52)	< 0.001	0.34(0.29–0.41)	< 0.001
Radiation								
Yes	Ref		Ref		Ref		Ref	
No/Unknown	1.19(1.11–1.28)	< 0.001	1.12(1.02–1.23)	0.019	1.13(1.04–1.22)	0.002	1.12(1.01–1.24)	0.038
Chemotherapy								
Yes	Ref		Ref		Ref		Ref	
No/Unknown	1.72(1.58–1.88)	< 0.001	1.75(1.49–2.05)	< 0.001	1.57(1.43–1.73)	< 0.001	1.79(1.51–2.12)	< 0.001

## Discussion

Gastric cancer is the fourth largest cancer threatening human health and survival, and with the improvement of diagnosis and treatment, its 5-years survival rate is increasing. Notably, adequate surgery stands as the sole curative treatment known thus far. However, owing to the aggressive nature of locally advanced gastric cancer, even after complete surgical resection, the incidence of postoperative recurrence and complications remains high, leading to a poor prognosis^17^. A multicenter phase III clinical trial in France demonstrated that the 5-years survival rate of surgical treatment alone is less than 25%, highlighting the imperative for combining other treatment modalities to enhance gastric cancer survival rates^18^. Conversely, adjuvant radiotherapy alone does not confer survival benefits in multidisciplinary comprehensive treatment approaches for gastric cancer^14^. Similar to the findings of existing studies, our retrospective analysis of different treatment modalities in patients with T3N1-3 gastric cancer found that multidisciplinary combination therapy (such as OP followed by adjuvant C/T with or without RT, CRRT followed by OP, and neoadjuvant C/T followed by OP and adjuvant C/T with or without RT) compared with OP and C/T and others alone provided a significant benefit for T3N1-3 gastric cancer patients with better 5-years survival. Among them, OP followed by adjuvant C/T with or without RT had the highest 5-years survival rate.

MAGIC and ACCORD-07, two randomized clinical trial studies, demonstrated that the perioperative chemotherapy group and the neoadjuvant chemotherapy group improved overall survival and progression-free survival (PFS) in patients with resectable adenocarcinoma of the stomach, lower esophagus, or gastroesophageal junction compared with the surgical treatment group, without increasing the rate of postoperative complications and the number of deaths within the first 30 days after surgery^18,19^.

Our study findings reveal that the OP followed by adjuvant C/T with or without RT treatment approach is associated with the highest 5-years survival rates. Similar conclusions were drawn from several trials. Both the ACTS-GC and CLASSIC trials demonstrated that postoperative chemotherapy improves overall survival (OS) and recurrence-free survival (RFS) in patients with gastric cancer after D2 gastrectomy compared with surgical treatment alone^20,21^. A meta-analysis enrolling 3838 patients after gastrectomy for gastric cancer revealed that postoperative adjuvant chemotherapy based on a fluorouracil regimen improved the 5yOS of gastric cancer compared to surgery alone^22^. The Intergroup 0116 (INT-0116) clinical trial demonstrated that the addition of adjuvant radiotherapy to chemotherapy based on fluorouracil and folinic acid significantly improved OS and RFS, and significantly reduced overall recurrence and local recurrence^23^. The ARTIST clinical trial illustrated the role of adjuvant radiochemotherapy in D2-resected gastric cancer patients, directly comparing it with fluoropyrimidine-platinum combination adjuvant chemotherapy. It was ultimately concluded that adjuvant chemotherapy and radiotherapy have similar disease-free survival (DFS) and OS in D2 resected GC and are equally beneficial in preventing recurrence. However, radiochemotherapy significantly improved DFS in patients with lymph node-positive disease and intestinal-type GC^24^.

In light of the above clinical trials and meta-analysis, a multidisciplinary treatment paradigm using surgery combined with CT or RT has become the standard treatment option for locally advanced gastric cancer. However, there is no single regimen that is universally accepted as the standard for multimodal treatment, and geographic disparities persist in the utilization of adjuvant chemotherapy, postoperative (adjuvant) radiotherapy, or perioperative chemotherapy (neoadjuvant). For example, in Asia adjuvant chemotherapy significantly improves the survival prognosis of patients with gastric cancer, whereas Western centers failed to achieve similar clinical outcomes. A joint study from the European Organization for Research and Treatment of Cancer (EORTC) Institutes and the International Cooperation on Cancer (ICCG) Institutes compared the efficacy of combined 5-fluorouracil with adriamycin or epirubicin and methotrexate with leucovorin rescue (FAMTX or FEMTX) with surgical treatment alone in terms of disease-free survival (DFS) and overall survival (OS). It was found that there was no significant difference in DFS and OS between the two groups, however, the survival rate was higher in the EORTC group compared to the ICCG group, which may be related to the more thorough surgery performed in the EORTC study^25^. Similarly, the results of two Italian phases III clinical trials, the GOIRC study and the GOIM 9602 study, showed no difference in OS or DFS between the adjuvant chemotherapy group (PELF regimen or ELFE regimen) and the surgery alone group^26,27^. Consistent with these findings, by comparing the 5yCSS of T3N1-3 gastric cancer patients treated with surgery alone versus adjuvant chemotherapy, we found that for patients with stage T3N1 surgery alone possessed a better 5yCSS, whereas for patients with stages T3N2 and T3N3 the 5yCSS between adjuvant chemotherapy and surgery alone was similar.

## Limitation

Our study has certain limitations. First, the geographical limitation of our study may not be representative. It cannot represent the efficacy of treatment programs for gastric cancer patients in other regions. Second, the use of a retrospective dataset for our study may cause bias in the results. Third, we were unable to obtain more detailed information about each treatment for each patient, such as the duration of systemic therapy, specific chemotherapeutic agents and dosages, time intervals between surgery and radiotherapy or chemotherapy, targeted therapies, immunotherapy, chemotherapeutic toxicity, and changes in quality of life. Fourth, there was a lack of information about the patient's gastrointestinal and hematologic history, physiologic function, and other influences that are most likely to change the patient's treatment plan and prognosis. Finally, the small sample size did not allow for more statistically significant results. Existing studies have shown some variation in the choice of treatment regimen across regions, and broader and more in-depth studies are needed in the future to determine the optimal treatment regimen and the survival benefit it provides. However, the SEER database covers approximately 30% of the U.S. population, and the results of future clinical studies will become increasingly compelling as the inclusion population continues to expand.

## Conclusion

A multimodal treatment regimen has a better survival prognosis than surgery and radiotherapy alone for patients with stage T3N1-3 gastric cancer. In particular, OP followed by adjuvant C/T with or without RT had the highest survival rate. This treatment regimen may be more suitable for non-Asian populations. In the future, multiregional, high-quality, large-scale, and long-term follow-up studies are needed to clarify the standard treatment strategy for patients with stage T3N1-3 gastric cancer.

### Supplementary Information

Supplementary Information.

## Data Availability

The dataset used in this study was obtained from the SEER 17 registry database of the National Cancer Institute (NCI) Surveillance, Epidemiology, and End Results (SEER) program. For instructions on accessing these data, please visit https://seer.cancer.gov/.
