# Supplementary material for: Comparison of different treatment strategies for T3N1-3 stage gastric cancer based on the SEER database
Source: Sci Rep. 2024 May 16;14:11210. doi: 10.1038/s41598-024-61904-8 (PMC11099173; doi:10.1038/s41598-024-61904-8)

**Comparison of different treatment strategies for T3N1-3 stage gastric cancer based on the SEER database**

**Supplementary Materials**

| **Section** | **Description** | **Pages** |
| --- | --- | --- |
| **Supplementary Tables** |  |  |
|  | **Table S1**  Five-year OS rate with different treatment options**.** | 2 |
|  | **Table S2**  Five-year CSS rate with different treatment options**.** | 2 |
|  | **Table S3**  Five-year OS rate with different surgical therapy**.** | 3 |
|  | **Table S4**  Five-year CSS rate with different surgical therapy**.** | 3 |
| **Supplementary Figures** |  |  |
|  | **Figure S1**  Kaplan-Meier survival curves show 5yOS rate for T3 gastric cancer patients with different N-stages based on different surgical therapy. | 4 |
|  | **Figure S2**  Kaplan-Meier survival curves show 5yCSS rate for T3 gastric cancer patients with different N-stages based on different surgical therapy. | 5 |

**Table S1** Five-year OS rate for T3N1-3 gastric cancer based on different treatment options.

|  | **Therapy Group** | | | | |  |
| --- | --- | --- | --- | --- | --- | --- |
| **Clinical Stage** | **1** | **2** | **3** | **4** | **5** | **P Value** |
| T3N1 | 0.336 | 0.473 | 0.390 | 0.458 | 0.191 | < 0.001 |
| T3N2 | 0.163 | 0.404 | 0.234 | 0.252 | 0.205 | < 0.001 |
| T3N3 | 0.050 | 0.261 | 0.205 | 0.194 | 0.105 | < 0.001 |

**Table S2** Five-year CSS rate for T3N1-3 gastric cancer based on different treatment options.

|  | **Therapy Group** | | | | |  |
| --- | --- | --- | --- | --- | --- | --- |
| **Clinical Stage** | **1** | **2** | **3** | **4** | **5** | **P Value** |
| T3N1 | 0.466 | 0.543 | 0,449 | 0.502 | 0.254 | < 0.001 |
| T3N2 | 0.256 | 0.484 | 0.286 | 0.287 | 0.249 | < 0.001 |
| T3N3 | 0.128 | 0.307 | 0.250 | 0.233 | 0.125 | < 0.001 |

**Table S3** Five-year OS rate for T3N1-3 gastric cancer based on different surgical therapy.

|  | **Surgical therapy** | | |  |
| --- | --- | --- | --- | --- |
| **Clinical Stage** | **No surgery** | **Partial gastrectomy** | **Total gastrectomy** | **P Value** |
| T3N1 | 0.114 | 0.442 | 0,367 | < 0.001 |
| T3N2 | 0.097 | 0.320 | 0.258 | < 0.001 |
| T3N3 | 0.083 | 0.197 | 0.160 | < 0.001 |

**Table S4** Five-year CSS rate for T3N1-3 gastric cancer based on different surgical therapy.

|  | **Surgical therapy** | | |  |
| --- | --- | --- | --- | --- |
| **Clinical Stage** | **No surgery** | **Partial gastrectomy** | **Total gastrectomy** | **P Value** |
| T3N1 | 0.155 | 0.526 | 0,429 | < 0.001 |
| T3N2 | 0.114 | 0.379 | 0.342 | < 0.001 |
| T3N3 | 0.113 | 0.252 | 0.201 | < 0.001 |

**Figure S1** Kaplan-Meier survival curves show 5yOS rate for T3 gastric cancer patients with different N-stages based on different surgical therapy. (A)T3N1, (B)T3N2, (C)T3N3, (D)T3N1-3.

A B


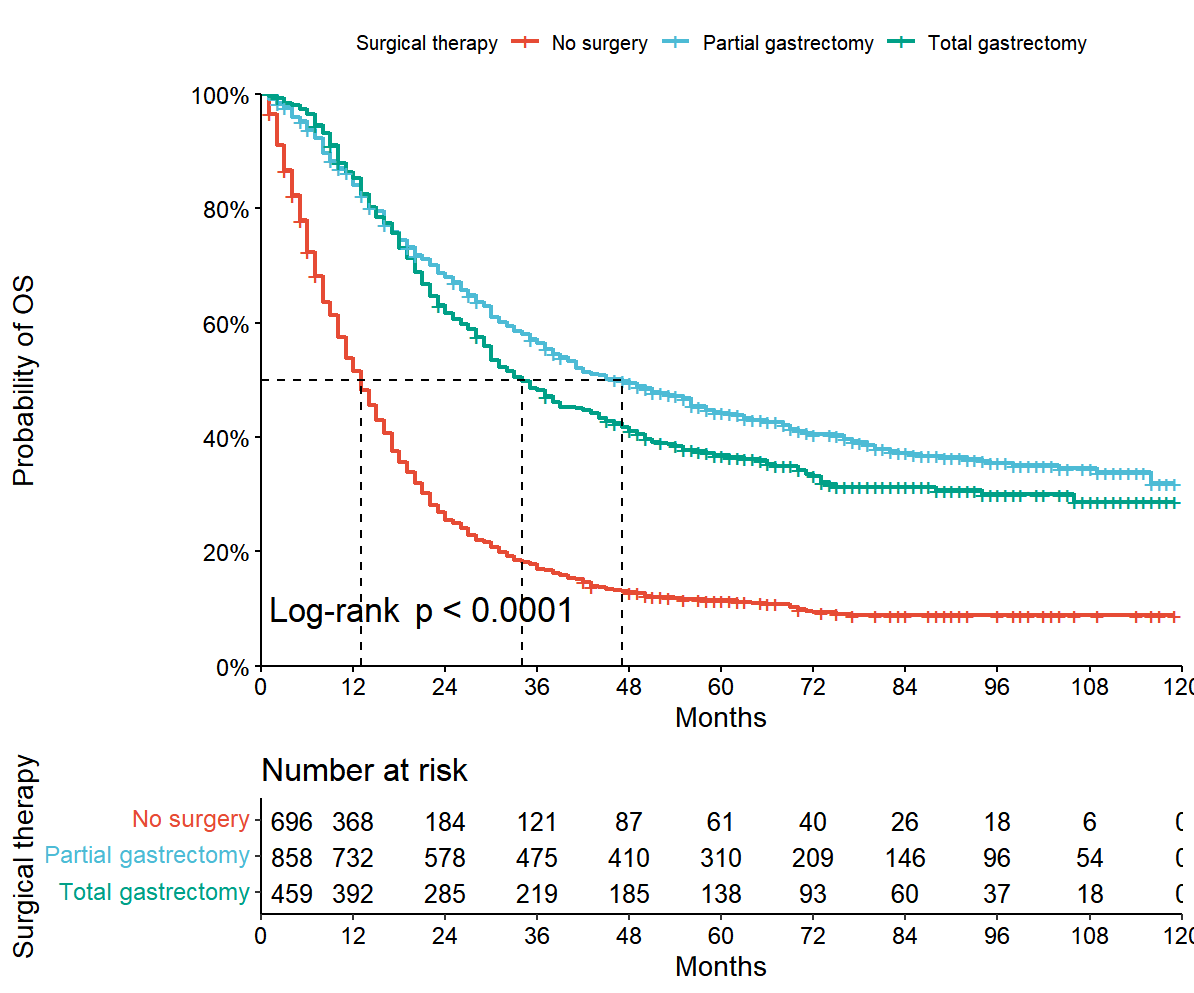

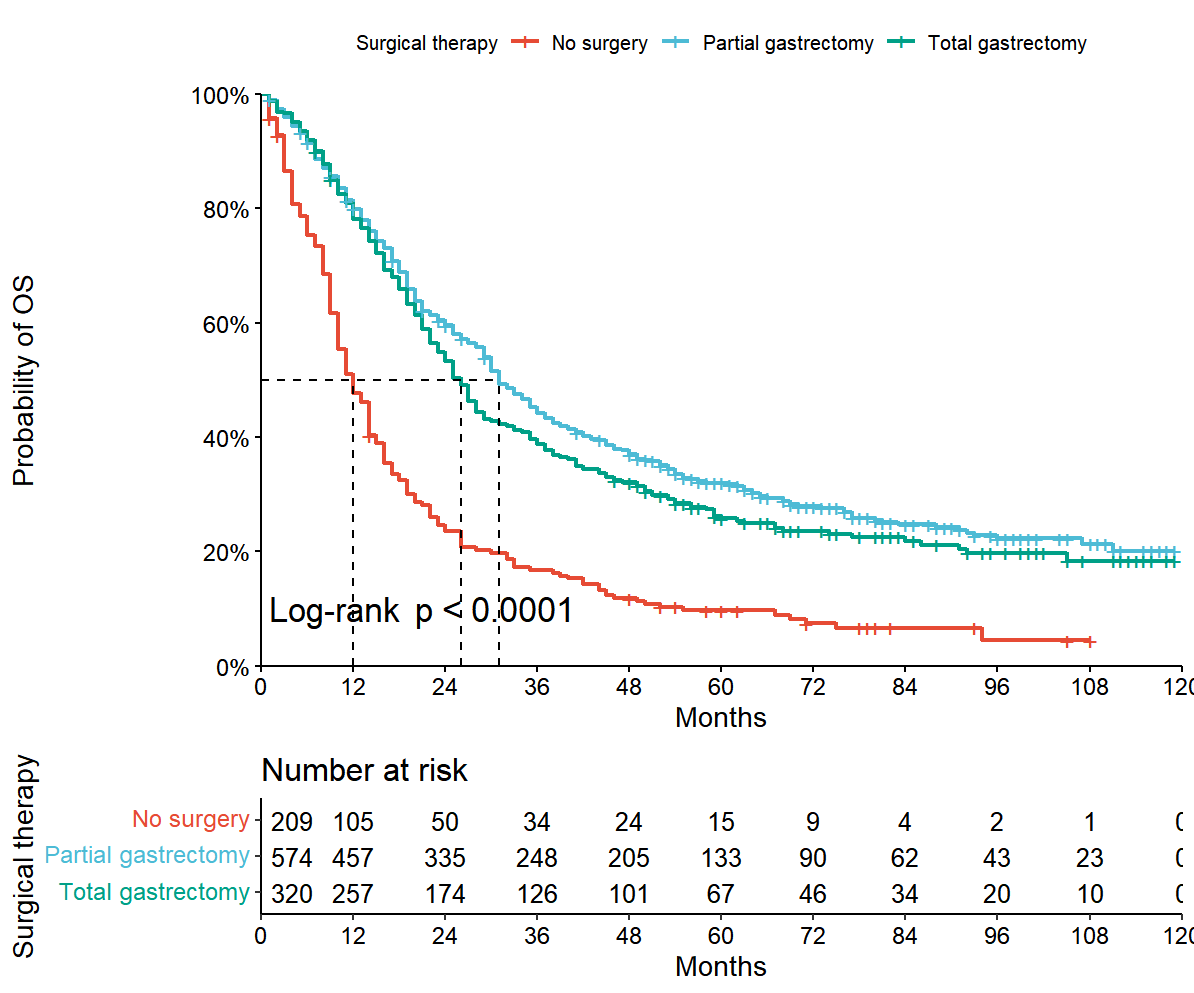


C D


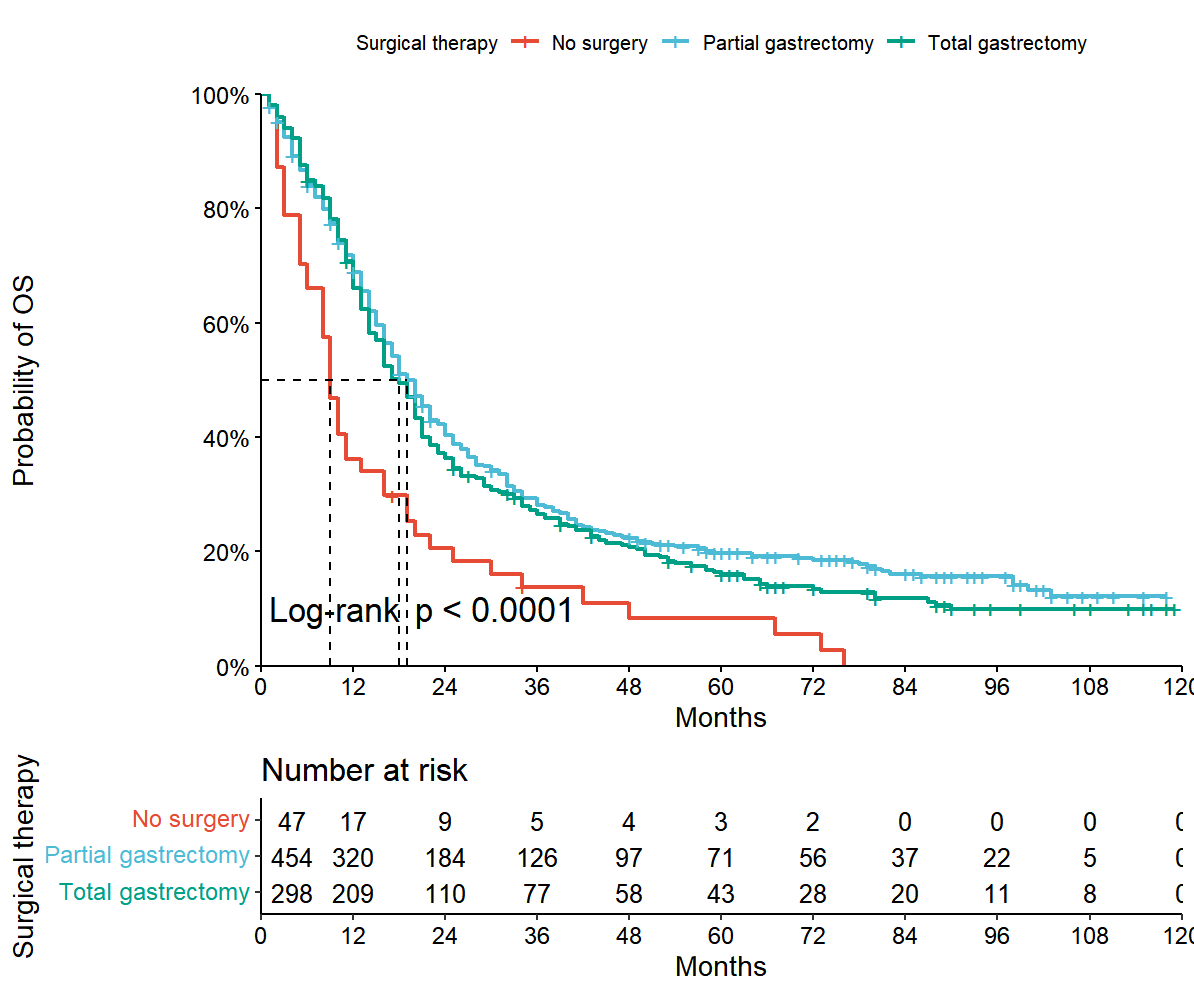

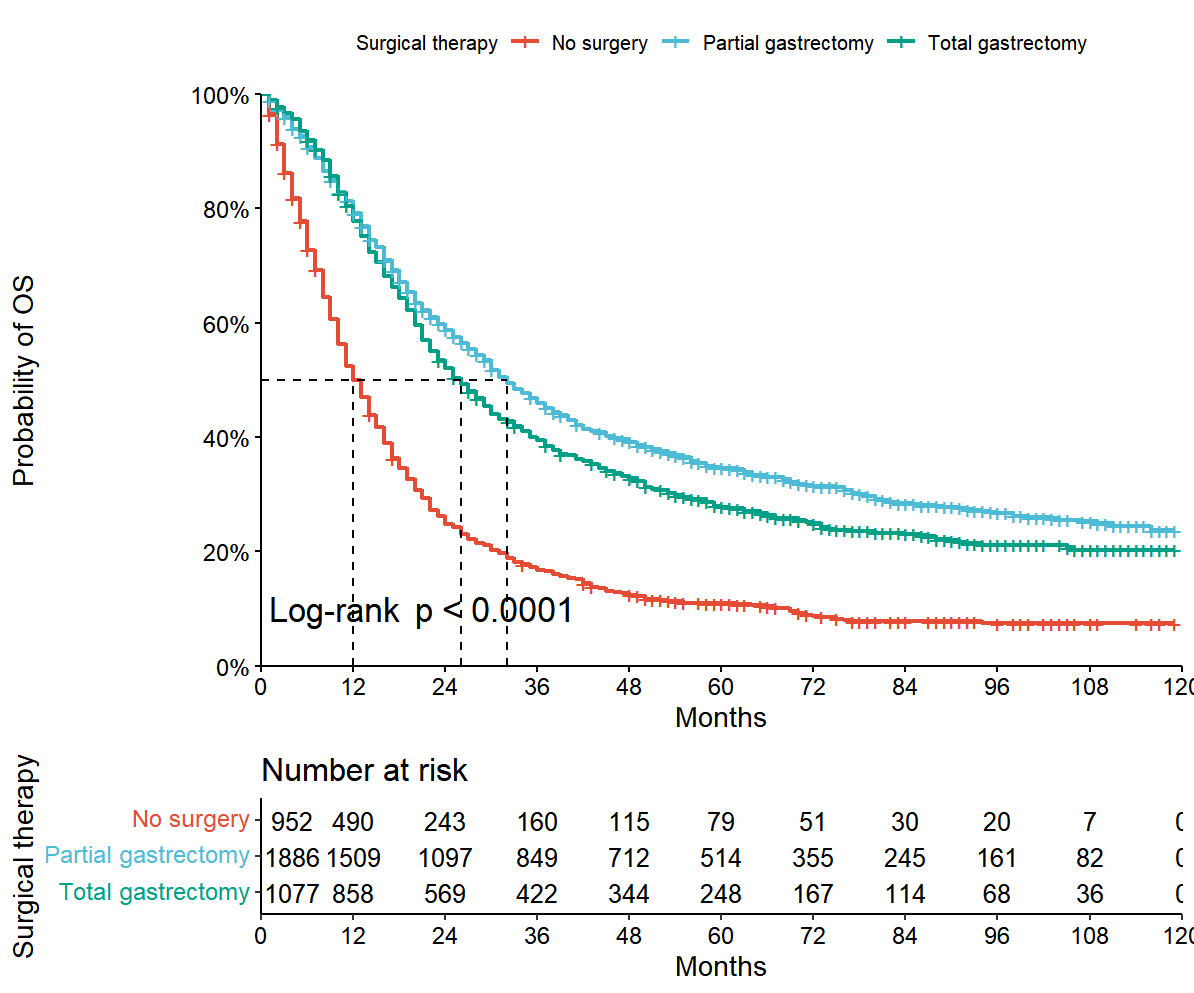


**Figure S2** Kaplan-Meier survival curves show 5yCSS rate for T3 gastric cancer patients with different N-stages based on different surgical therapy. (A)T3N1, (B)T3N2, (C)T3N3, (D)T3N1-3.

A B


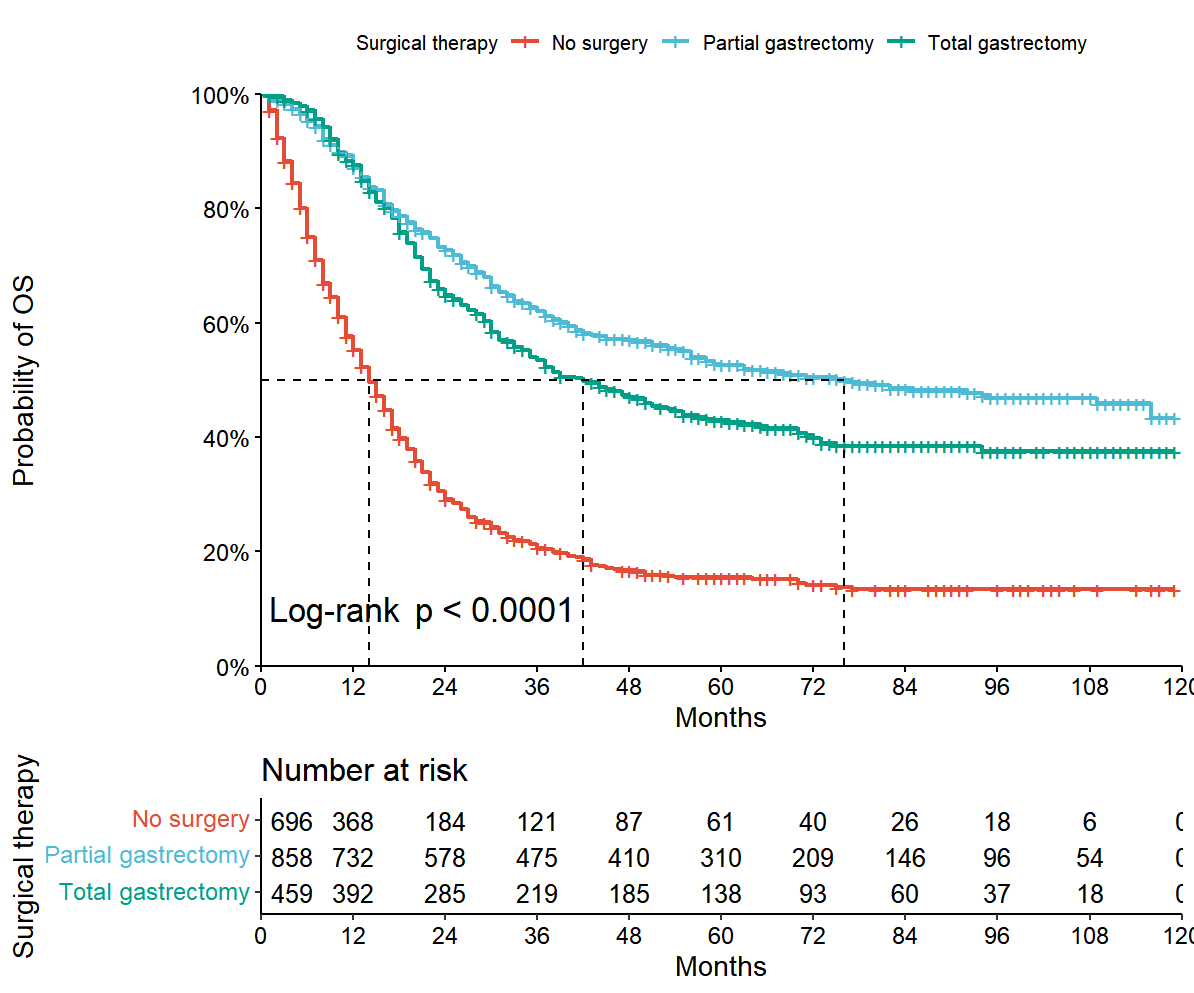

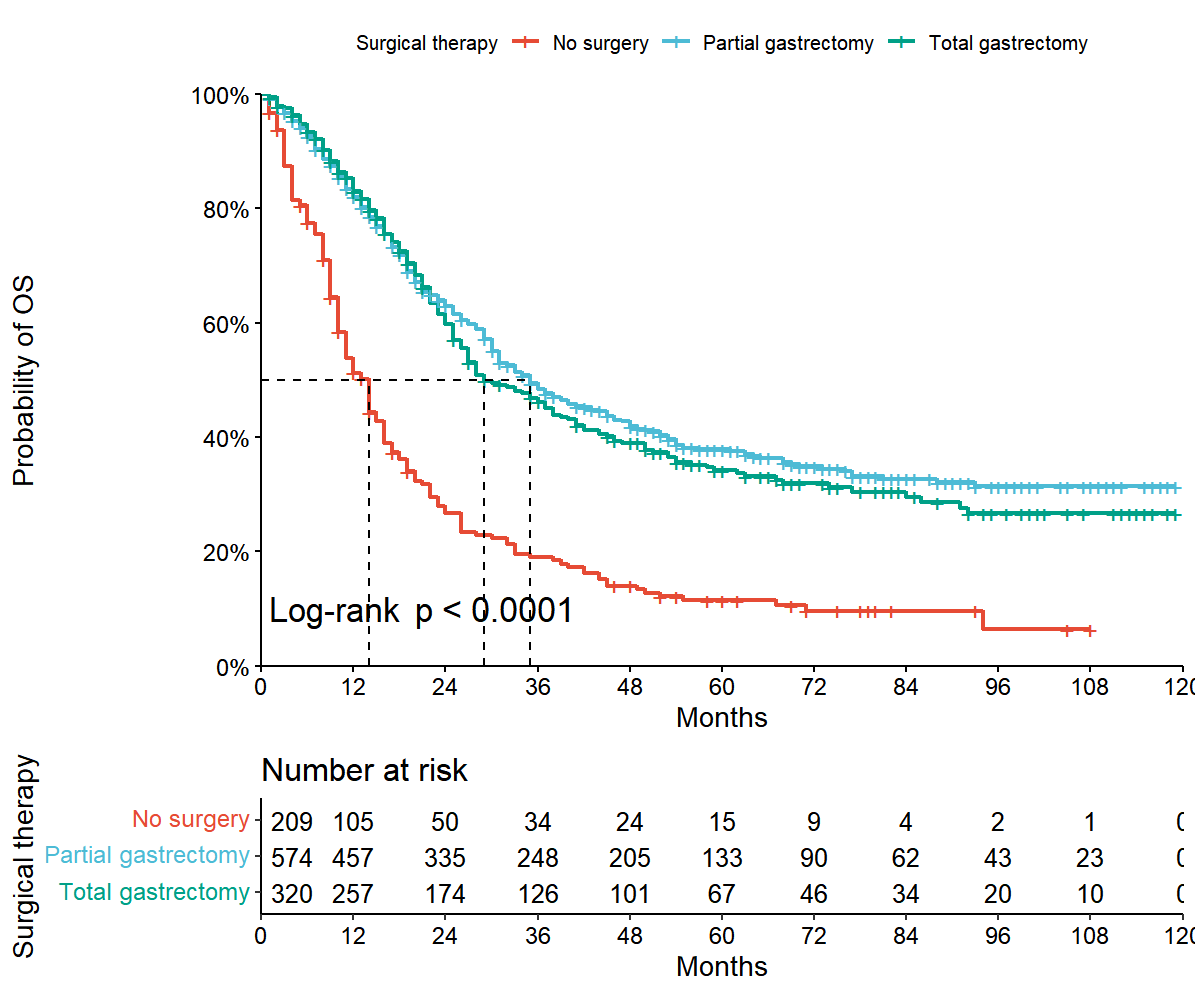


C D


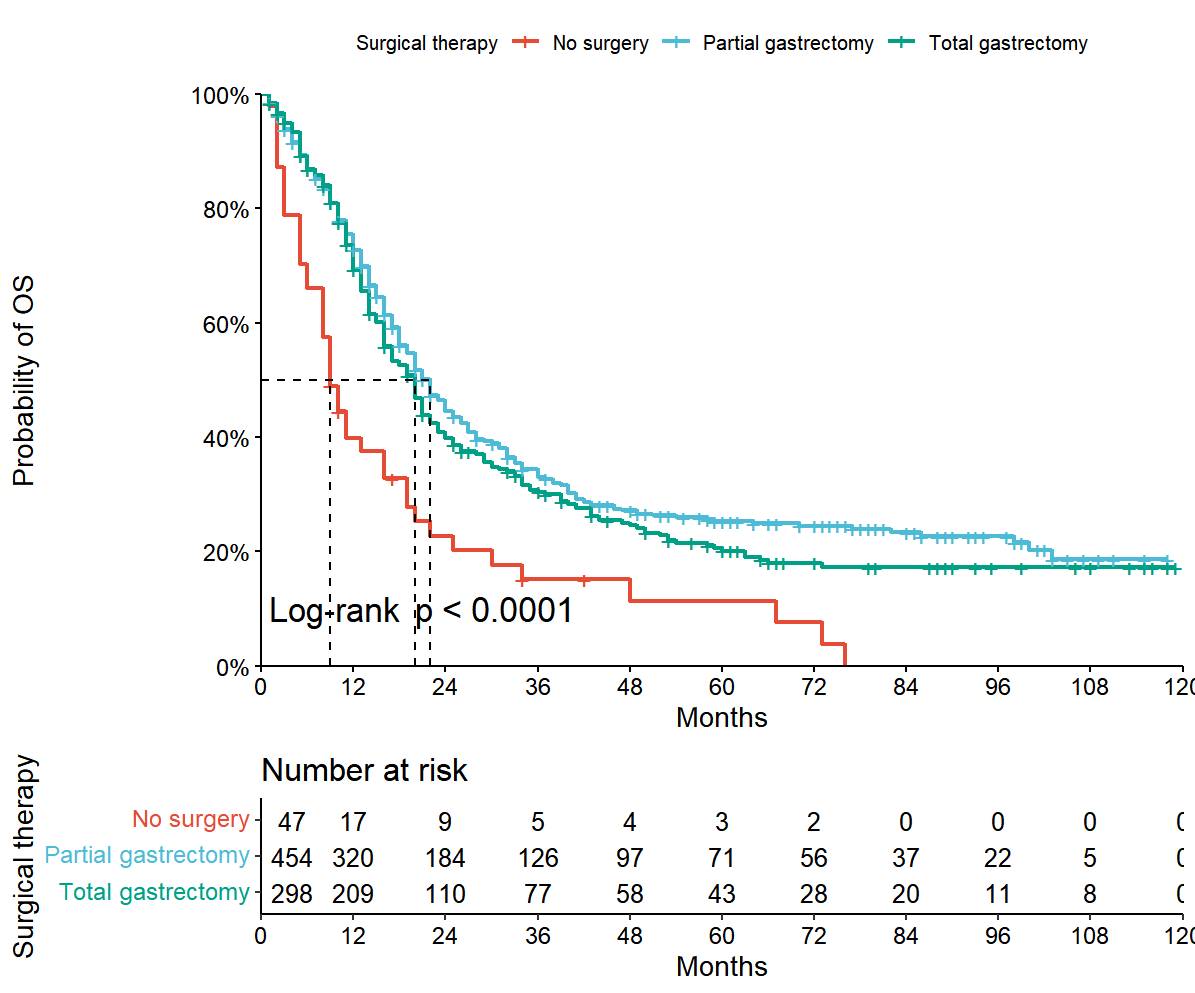

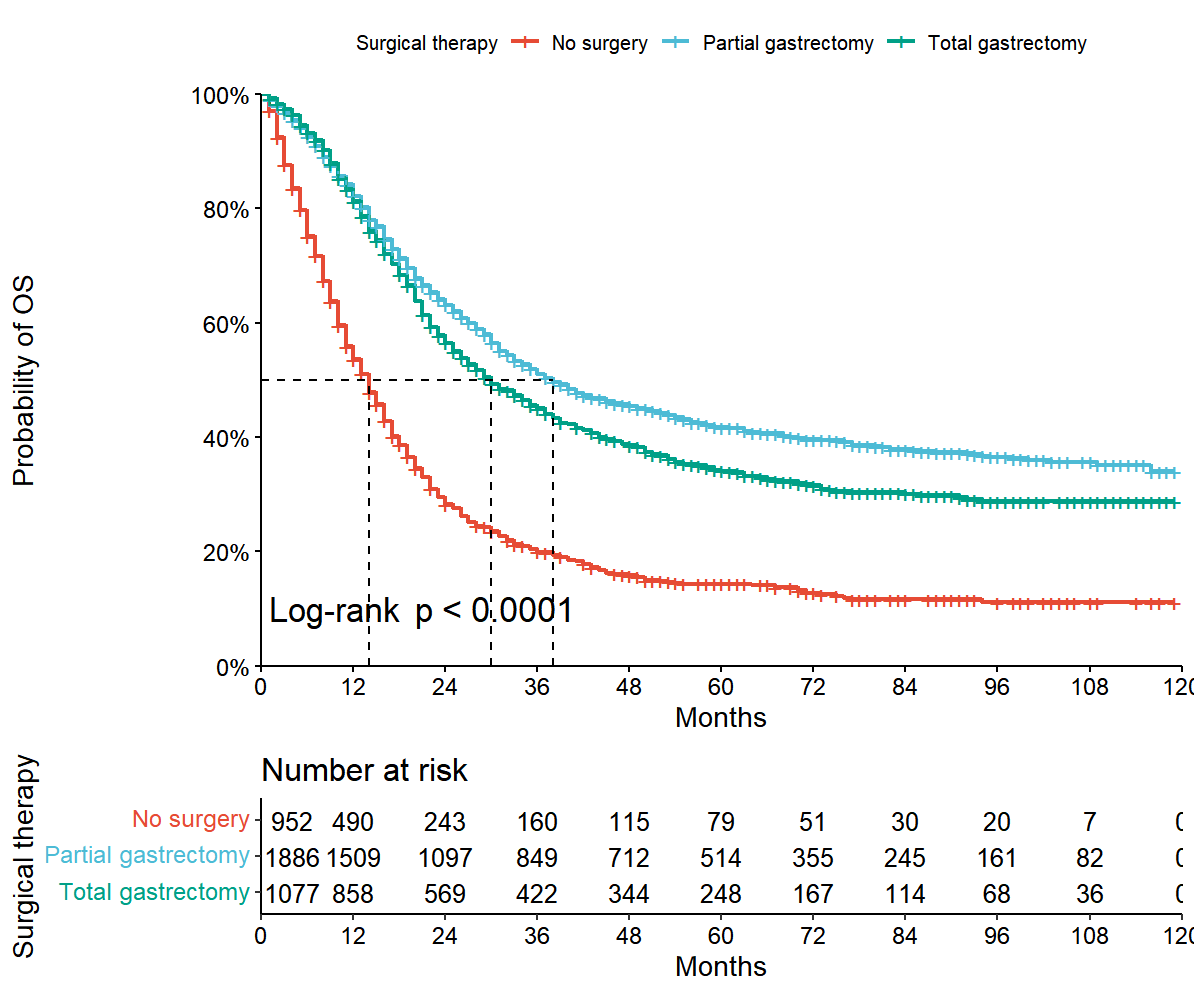

Supplement: Supplementary file 1 — Supplementary Information. [file 41598_2024_61904_MOESM1_ESM.docx]
